# Surface Modification of Ti-6Al-4V Alloy by Electrical Discharge Coating Process Using Partially Sintered Ti-Nb Electrode

**DOI:** 10.3390/ma12071006

**Published:** 2019-03-27

**Authors:** Chander Prakash, Sunpreet Singh, Catalin Iulian Pruncu, Vinod Mishra, Grzegorz Królczyk, Danil Yurievich Pimenov, Alokesh Pramanik

**Affiliations:** 1 School of Mechanical Engineering, Lovely Professional University, Phagwara 144411, India; chander.mechengg@gmail.com; 2 Mechanical Engineering, Imperial College London, Exhibition Rd., SW7 2AZ London, UK; c.pruncu@imperial.ac.uk; 3 Optical Devices and Systems, Central Scientific Instruments Organization, Chandigarh 160030, India; vnd.mshr@gmail.com; 4 Faculty of Mechanical Engineering, Opole University of Technology, 76 Proszkowska St., Opole 45-758, Poland; g.krolczyk@po.opole.pl; 5 Department of Automated Mechanical Engineering, South Ural State University, Lenin Prosp. 76, Chelyabinsk 454080, Russia; danil_u@rambler.ru; 6 Department of Mechanical Engineering, Curtin University, Bentley, Perth 6102, WA, Australia; alokesh.pramanik@curtin.edu.au

**Keywords:** Ti-6Al-4V, alloy, EDC, microcracks, microhardness, adhesion strength

## Abstract

In the present research, a composite layer of TiO_2_-TiC-NbO-NbC was coated on the Ti-64 alloy using two different methods (i.e., the electric discharge coating (EDC) and electric discharge machining processes) while the Nb powder were mixed in dielectric fluid. The effect produced on the machined surfaces by both processes was reported. The influence of Nb-concentration along with the EDC key parameters (Ip and Ton) on the coated surface integrity such as surface topography, micro-cracks, coating layer thickness, coating deposition, micro-hardness has been evaluated as well. It has been noticed that in the EDC process the high peak current and high Nb-powder concentration allow improvement in the material migration, and a crack-free thick layer (215 μm) on the workpiece surface is deposited. The presence of various oxides and carbides on the coated surface further enhanced the mechanical properties, especially, the wear resistance, corrosion resistance and bioactivity. The surface hardness of the coated layer is increased from 365 HV to 1465 HV. Furthermore, the coated layer reveals a higher adhesion strength (~118 N), which permits to enhance the wear resistance of the Ti-64 alloy. This proposed technology allows modification of the mechanical properties and surface characteristics according to an orthopedic implant’s requirements.

## 1. Introduction

Among all metallic biomaterials, Ti-6Al-4V (Ti-64) alloy is most widely used material for biomedical applications to fabricate implants and surgical instruments due to its unique feature of great mechanical properties and excellent biocompatibility [1,2]. The Ti-64 alloy has a Ti-oxide bio-inert layer, which has low hardness and poor wear resistance [3]. In order to improve its surface properties and characteristics, a number of surface treatment/modification processes were reported [4]. In the current research scenario, the electrical discharge machining (EDM) process is the only non-conventional machining process, which permits effective manufacturing of Ti-64 alloy that is hard to cut [5]. Moreover, EDM produce a bio-compatible layer on the surface, which promotes a higher surface hardness and better corrosion resistance of Ti-64 alloy [6]. Peng et al. used EDM to machine the Ti-64 alloy and tuned the surface characteristics as required for the osseiointegration process [7]. The nanoporous layer has been synthesized by EDM, which promotes cell adhesion and growth [8]. Bin et al. investigated the effect of EDM to improve the surface hardness, wear resistance, corrosion resistance, and bioactivity of Ti-64 alloy [9]. The application of EDM for coating/deposition/alloying has been extended by altering the tool electrode polarity [10,11], powder metallurgical (P/M) prepared from green compact tool electrode [12,13,14,15], and powder mixed dielectric [16,17]. Most researchers use powder mixed dielectric to make deposition into a workpiece surface [18,19]. Prakash et al. investigated the effect of Si-mixed EDM to alter the surface characteristics that allows improving the bio-compatibility, corrosion, and wear resistance properties of a specially designed Ti-35Nb-7Ta-5Zr (β-phase) for orthopaedic applications [20]. Furthermore, multi-objective optimization of Si-mixed EDM by the Non-dominated Sorting Genetic Algorithm-II (NSGA-II) were used to synthesize the bio-mimetic surface [21]. The powder mixed electric discharge machining (PMEDM) was also utilized to enhance the fatigue performance and bone-implant interface [22,23]. Xie et al. reported that the surface hardness of 45-C steel has been increased from 415 to 1420 HV using graphite-mixed EDM [24]. Arun et al. synthesized a hard-layer of Ni-W coating on tool-steel by Ni &W—mixed EDM in order to improve the tribological performance [25]. Ekmekci et al. reported that a hydroxyapatite (HA) enriched bioceramic layer can be successfully deposited on Ti-64 surface using HA-mixed EDM process [26]. Ou and Wang used EDC to deposit a HA-enriched layer on Ti alloy in order to enhance the biocompatibility of base material [27]. Prakash et al. deposited HA-enriched biomimetic porous layer on the Ti-35Nb-7Ta-5Zr (β-phase) surface to enhance the bio-mechanical and corrosion integrity [28]. Recently, Prakash et al. uncovered the ability of HA-mixed EDM process to enhance the mechanical, corrosion, bioactivity of Mg-based biodegradable implants [29,30]. The EDM/PMEDM was utilized for surface modification of biomedical implants [31,32,33]. 

There is wide scope in additive mixed EDM process to deposit a bio-compatible layer for orthopaedic applications using a different type of additive mixed in dielectric fluid. To date, no research study has reported the application of niobium (Nb) powder mixed EDM for surface modification of Ti-alloy. As such, the present research investigates the effect of a multi-composite layer (i.e., of TiO_2_-TiC-NbC-NbO) deposited on the Ti-64 alloy surface using EDC process. The results gathered in this work prove that the surface of Ti-64 treated by Nb was enhanced in terms of bio-mechanical integrity and wear resistance.

## 2. Materials and Methods

### 2.1. Characterization of Sintered Ti-Nb Alloy and Machined Surface by Electric Discharge Coating (EDC) Process

**Figure 1:**
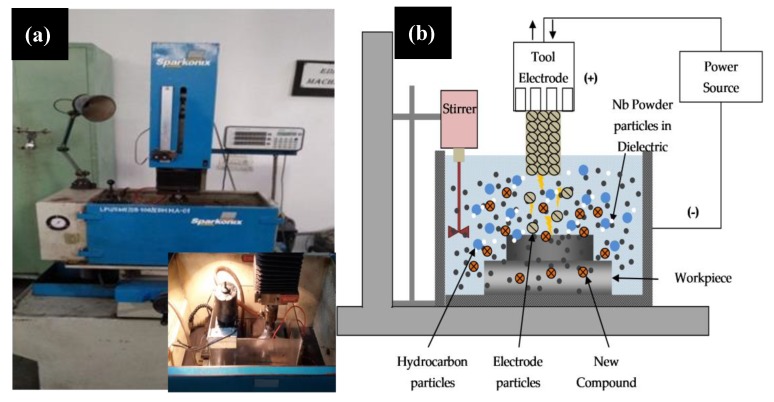
(**a**) Experimental set-up of EDC process and (**b**) schematic representation of EDC process.

Ti-6Al-4V extra low interstitials alloy (Ti-64 ELI) was used as the workpiece material, provided by Titanium-India, Mumbai. The samples of 10 mm width, 10 mm length, and 5 mm thickness were cut from the as-received cast ingot. The surfaces of the cut samples were polished up to Ra ~ 0.5 μm. The surface modification of Ti64-ELI alloy was carried out by depositing a thin coating layer by the electric discharge coating process while the Nb-powder particles were mixed in a dielectric fluid. Here, were used a commercially pure-Ti tool electrode. Table 1 shows details of experimental conditions. Figure 1a schematically shows the experimental set-up of the EDC process whereas Figure 1b schematically presents the main mechanism of deposition that includes the coating layer of oxides and carbides in the EDC process. A partially sintered tool electrode was used for the coating process.

### 2.2. Development of Partially Sintered Tool Electrode for EDC Process

**Figure 2:**
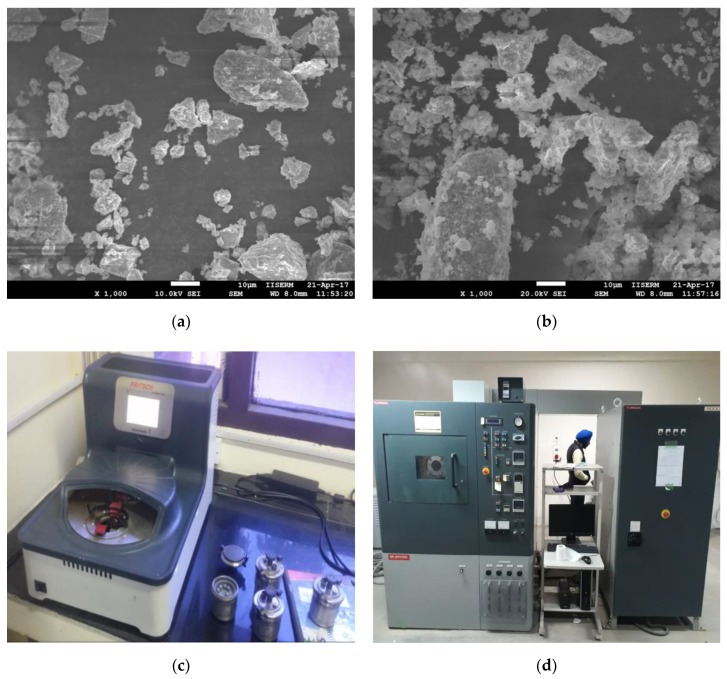
(**a**,**b**) Scanning electron microscope (SEM) micrograph showing the morphology of titanium and niobium powders; (**c**) high energy planetary ball mill; and (**d**) spark plasma sintering (SPS) machine.

**Figure 3:**
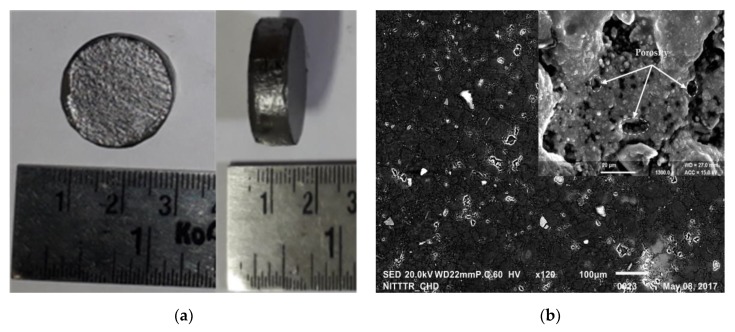

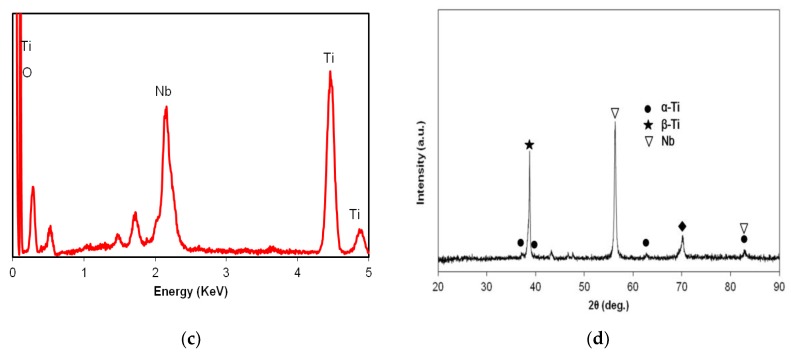
(**a**) Sintered Ti-Nb alloy; (**b**) SEM micrograph showing the morphology; (**c**) EDS spectrum; (**d**) X-ray diffraction (XRD) pattern of Ti-Nb alloy.

The partially sintered Ti-Nb tool electrode was developed by spark plasma sintering process. Titanium (>99.9% purity, 45µm) and niobium (99.9% purity, 45 µm) were procured from N.B. enterprises, Bilaspur, India. Here we present the main steps followed for the fabrication of Ti-Nb alloy: (i) Ti and Nb were mixed 50:50 weight percentages. The mixture of Ti-Nb was developed in a planetary ball mill with tungsten balls (ball to powder ration kept 5:1) at a rotational speed of 200 rpm for 8 h. Figure 2 shows the shape and size of Ti and Nb powders. (ii) The as-blended powder mixture was consolidated via the spark plasma sintering (SPS) method in a graphite die at 800 °C sintering temperature using a heating rate of 100 °C (holding time 15 min) [34]. This was performed under vacuum conditions while the uniaxial pressure was kept constant at 50 MPa. Figure 3 shows a photograph of the SPS machine available at the Indian Institute of Technology, Roorkee, and the red hot sample under vacuum condition during the process. 

### 2.3. Characterization of Sintered Ti-Nb Alloy and Machined Surface by EDC Process 

The topology and morphology of coated surface were analyzed with a field emission scanning electron microscopy (FE-SEM; JEOL 7600F; JEOL Inc., Peabody, MA, USA). The phase and element constituents in the as-synthesized composites were determined by the X-ray diffraction (XRD) technique (XRD; X’pert-PRO, PANalytical, Almelo Inc., Almelo, Netherlands) and energy dispersive spectroscopy (EDS) (FE-SEM; JEOL 7600F; JEOL Inc., Peabody, MA, USA), respectively. XRD pattern peaks were analyzed according to the database of the Joint Committee on Powder Diffraction Standards (JCPDS), to identify the phases formed in the coated surface. The thickness of the coating layer (CLT) was measured by investigating the cross-section of the modified specimens [19,20]. For the CLT measurements, the test surfaces were well polished and prepared using adequate grinding and polishing methods according to the ASTM standard (ASTM-E384-11) [19]. The surface hardness of the coated layer was measured with a Shimadzu HMV-G21 (Vickers) micro-hardness tester (HMV-G21ST, SHIMADZU, Japan), using an indenting load of 0.49 N during 15 s dwell time. The cross-sectional surface of the samples was used to measure the microhardness values. The adhesive strength between the coating and the substrate was measured with a scratch tester (TR-102, DUCOM, Bengaluru, India). The loading force was increased up to 150 N by using a loading rate of 25 N/mm and the scratch length was 4 mm.

## 3. Results and Discussion

This section is divided into two subsections. The first section provides a concise and precise description of the results from the fabrication of Ti-Nb alloy using the SPS method. The second section provides the experimental results obtained by EDC technique, their interpretation as well as the experimental conclusions formulated from the analysis.

### 3.1. Microstructure, Morphology, and Chemical Composition of Sintered Ti-Nb Electrode

Figure 3 shows details of the sample produced, its microstructure and phase composition of a partially sintered Ti-Nb alloy obtained with the SPS technique, which was further used in the EDC process. Figure 3a shows the photograph of sintered Ti-Nb alloy that is a compacted sample of 20 mm diameter and thickness of 6 mm. Figure 3b shows the SEM micrographs of the sintered sample obtained at a sintered temperature of 700 °C. These micrograph indicates the presence of porosities in the sample which is a sign of a sample partially sintered. The percentage of porosities in the sintred Ti-Nb alloy is was found ~25%. The porosities are uniformly distributed having the pore size of 5–15 µm. Furthermore, the chemical reaction between the alloy elements generated large amounts of gases that leads to the creation of porosity in the structure. At higher magnification (5000×), the presence of α-Ti and β-Nb phases can be identified clearly; the α-Ti is a dark phase and β-Nb is the brighter phase. The dissolution of Nb is incomplete, and the equiaxed microstructure of β-Nb uniformly distributed and surrounded by α-Ti matrix. Figure 3c shows the associated EDS spectrum of the sintered Ti-Nb alloy. The EDS spectrum confirms the presence of Ti and Nb elements. Apart from the presence of base metal elements, the element O (possible oxides) was noticed as well on the surface, which is a common observation in the SPS-treated surface [35,36,37,38,39,40]. Figure 3d shows the associated XRD pattern of the sintered Ti-Nb alloy and endorses the phase composition of the sample at the different sintering temperature. The XRD pattern of sintered Ti-Nb alloy revealed the presence of α’ phase (Ti), together with β bcc phase (Nb) weak peaks, partially overlapped on α’ peaks. It can be seen that the β-phase is the major phase in the sintered samples and the intensity of peaks of β-phase increases as the sintering temperature increases. This is because at lower sintering temperature the sample consists alpha phase, whereas, at higher sintering temperature only the beta phase is produced with a minor amount of alpha phase. Therefore, the peaks of sintered sample are high at higher temperatures associated with the presence of the beta phase. The partially sintered porous Ti-Nb alloy was further utilized as an electrode material for the surface modification of the Ti-6Al-4V alloy by depositing a layer of Ti-Nb by electric discharge coating process to enhance its surface and mechanical properties. The results of the EDC using partially sintered porous Ti-Nb alloy were reported in detail within the next section. The surface topography, elemental composition, coating thickness, material deposition, surface microhardness and adhesion of the coated surface has been simulated with the use of partially sintered porous Ti-Nb alloy as the tool electrode.

### 3.2. Morphology, Chemical Composition, and Microhardness of EDC-Treated Surface

**Figure 4:**
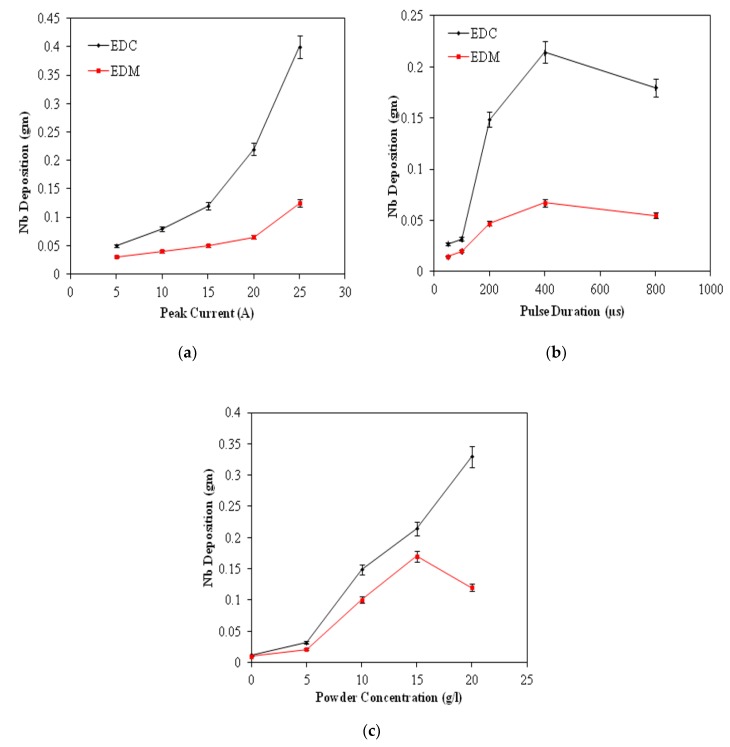
Variation of Nb deposition coating with (**a**) peak current, (**b**) pulse duration, and (**c**) Nb Powder concentration.

The effect of process parameters on the Nb deposition, micro-cracks, coating thickness and surface chemistry has been investigated by varying the key parameters of the EDC process (Ip, Ton, and Pc). The amount of Nb deposition on the workpiece surface was measured by EDS analysis. In the present study, the effect of input process parameters on the deposition of weight percentage of Nb powder has been evaluated. Figure 4 shows the variation of process parameters on the deposition of weight percentage of Nb powder in EDM and EDC. As the peak current increases the weight percentage of Nb powder deposition on the workpiece surface first increases up to 20 A. If the peak current increases further, the weight percentage of Nb powder deposition on the workpiece surface start decreases (Figure 4a). The trend for the variation of deposition of Nb on the Ti-64 surface with respect to peak current is the same in both cases, but much less Nb has been deposited on Ti-64 surface as compared to EDC. The maximum Nb (0.42 gm) was deposited at 25A peak current. This is attributed to the increases of peak current and the discharge energy generated which resulted in large exploratory pressure on the dielectric fluid causing migration and deposition of Nb powder towards workpiece surface. Figure 4b shows the variation of weight percentage of Nb powder deposition on the workpiece surface in respect to the pulse duration. The weight percentage of Nb deposition on the workpiece surface first increases with pulse-duration (up to 400 µs) and then start decreasing once the pulse-duration increases further. The explanation agrees with the peak current one, because with the increase in pulse duration the duration of discharge energy in the machining area increases which maintains pressure on the dielectric fluid for the continuous migration and deposition of Nb powder on the workpiece surface. On the other hand, at high pulse duration, the discharge energy generated in a very large exploratory pressure results in a spattering of the molten pool; thus less weight percentage of Nb powder deposition on the workpiece surface. The trend for the variation of deposition of Nb on the Ti-64 surface with respect to pulse duration is the same for both cases, but much less Nb has been deposited compared to EDC. The maximum Nb was deposited such as 0.22 gm at 400 µs of pulse duration. Figure 4c shows the variation of weight percentage of Nb powder deposition on the workpiece surface in respect to Nb powder concentration. As the Nb powder concentration increases, the weight percentage of Nb powder deposition on the workpiece surface increases. This is because, with the increase in Nb-concentration, the Nb-powder particles and eroded-debris are unable to flush out, and are charged due to the ionization of dielectric fluid. As a result, these spark products migrate toward the workpiece surface. The weight percentage of Nb powder deposition on the workpiece surface increases with the increases of the coating thickness. The maximum Nb was deposited 0.34 gm at 20 g/L of Nb powder concentration. 

**Figure 5:**
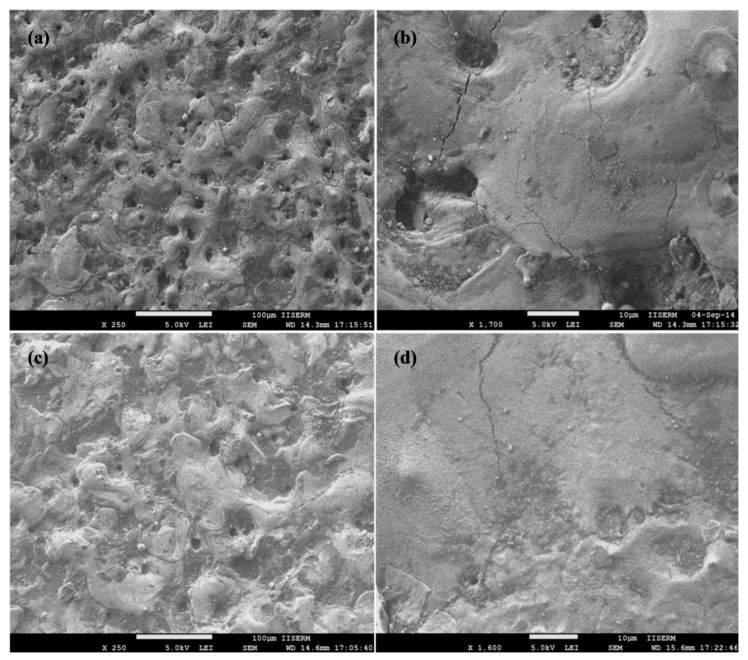
SEM morphology of Ti-6Al-4V alloy after (**a**,**b**) EDM-treatment and (**c**,**d**) EDC-treatment.

Figure 5 shows the surface morphology of the Ti-6Al-4V alloy surface after EDM (tool electrode positive polarity) and EDC (tool electrode negative polarity) setting the peak current = 10 A, pulse duration = 100 µs, duty cycle = 8%, and machining time = 15 min. Many craters resulted from electoral sparks were found on EDM-and EDC-treated surface, but both surfaces have different morphology, which can be observed in Figure 5a,c, respectively. At higher magnification (1700×), a higher density of surface micro-cracks along with high ridges of redeposited molten metal, globules, and pock marks have been identified on the EDM-treated surface, which results in poor surface quality (Figure 5b). On the other hand, the EDC-treated surface shows the micro-cracks but smooth surface as compared to the EDM-treated surface. This is because, in the EDM process, the heat energy is high at the workpiece surface which leads to the removal of workpiece material in the form of deep and wide craters and results in high ridges of redeposited material [18,22,23]. On the other hand, in the case of EDC, the heat energy is high at tool electrode side and low at workpiece side. As a consequence less melting of the workpiece material as compared to the EDM process and results in flat ridges of redeposited material. The results agree with the findings of the previous results reported [25,26]. The EDC-treated surface has less intensity of crack, free from pock marks, globules and Ti and Nb particles in the form of a coated layer can be clearly seen (Figure 5d). This is because during the EDC process, the tool electrode material and degraded carbon form the dielectric fluid, and fill the spaces of micro-cracks, micro-sized craters, and voids. As a result, a layer of oxides and carbides on the machined surface is produced that allows the elimination of micro-cracks.

**Figure 6:**
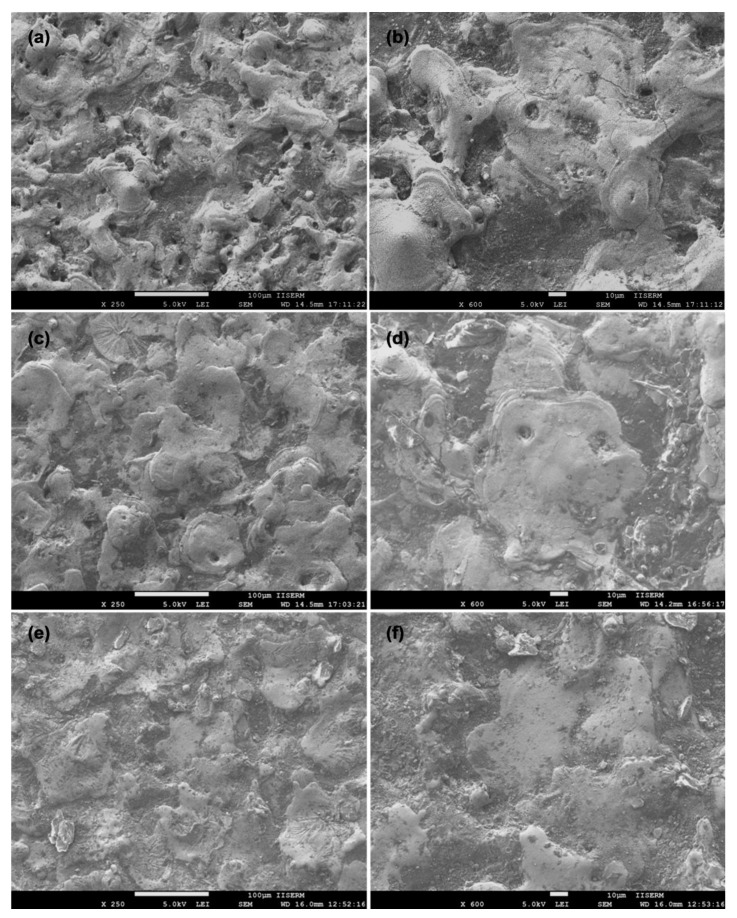
SEM morphology of Ti-6Al-4V alloy after EDC treatment at Nb concentration of (**a**,**b**) 10 g/L; (**c**,**d**) 15 g/L and (**e**,**f**) 20 g/L.

Figure 6 shows the surface morphology of the Ti-6Al-4V alloy surface after EDC treatment setting the machining condition of peak current = 10 A, pulse duration = 400 µs, duty cycle = 8%, and machining time = 15 min with Nb powder concentration of 10, 15 g/L and 20 g/L, respectively. Relative to the EDM-treated surface, a very flat and smooth surface is observed but only few micro-cracks and micro-pits still exist on the surface at 10 g/L Nb powder concentration, as can be seen in Figure 6a,b. When Nb concentration increased to 15 g/L, a crack and pit-free smooth surface was observed. Apart from this, a layer of oxides and carbides in a higher proportion has been identified, as can be seen in Figure 6c,d. When Nb concentration increased to 20 g/L, a crack and pit-free surface together with a layer of oxides and carbides in a higher proportion was identified that is depicted in Figure 6e,f. This is because, the Nb powder concentration increases the formation of compacted surface cracks while a layer of oxides and carbides (Ti-C, Ti-O, Nb-C, and Nb-O) is formed. Janmanee et al. reported the similar findings in their research for the reduction of micro-cracks on the EDC-treated surface and demonstrated how a layer of WC-Co and Ti-C formed on the machined surface allows the cracks and voids to be filled; causing a reduction in surface cracks [10]. In previous studies, it has been reported that the powder addition in the dielectric fluid significantly reduced the formation of surface cracks [17,18,19,20].

**Figure 7:**
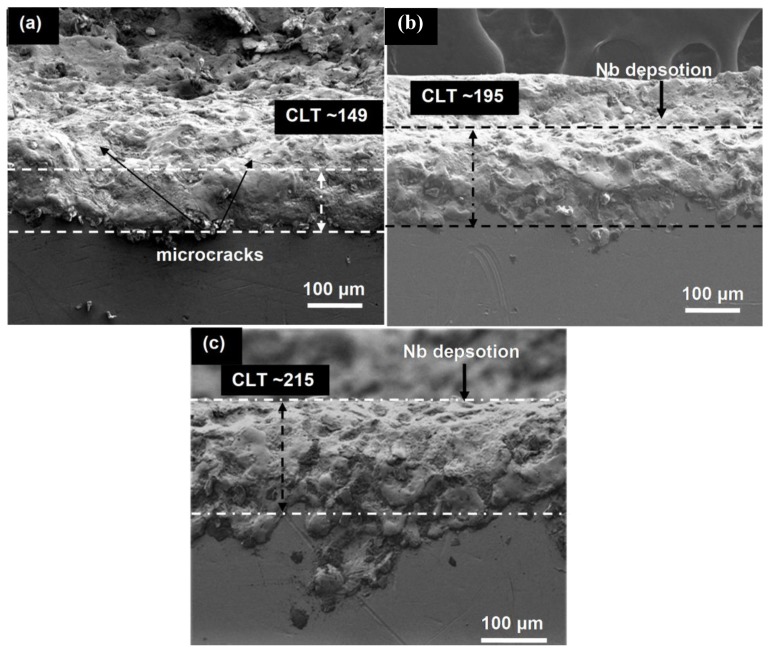
Cross section morphology of coating layer on Ti-6Al-4V alloy: (**a**) 149 µm thickness after EDM-treatment, (**b**) 195 µm thickness after EDC-treatment, and (**c**) 215 µm thickness after EDC-treatment.

Figure 7 shows the cross section of a micrograph of the coating layer on Ti-6Al-4V alloy after EDM and EDC treatment setting the condition of peak current = 10 A, long pulse duration = 400 µs, duty cycle = 8%, and machining time = 15 min. It can be observed that the thickness of recast layer on Ti-6Al-4V alloy after being EDM-treated was about 149 µm with many surface defects. Surface micro-cracks and resolidified drops of the molten material can be seen on the EDM-treated surface. The re-solidified material has poor bonding and loosely connected thus there is a risk of their loosening and particles may cause considerable danger since they can penetrate between articulating parts of the joint and damage them. On the other hand, a very smooth and thick layer of coating was observed on the EDC-treated surface when Nb concentration was 10 g/L. The thickness of the coated layer was measured ~195 µm which was thicker than that of the EDM-treated surface, but was defect free. When Nb concentration is 20 g/L. The thickness of the coated layer was measured ~215 µm which was thicker than that of the EDM-treated surface, but still defect free. The loose surface particles are not observed on the EDC-treated surface and excellent metallurgical bonding of re-solidified material with base material were seen, which permits the properties of the modified surface to be enhanced. The deposition of the resolidified material on the workpiece surface has a direct relation with peak current, pulse duration, and powder concentration. The higher peak current and pulse duration generates higher discharge energy which results in repelled both debris and suspended powder particles towards the workpiece surface.

**Figure 8:**
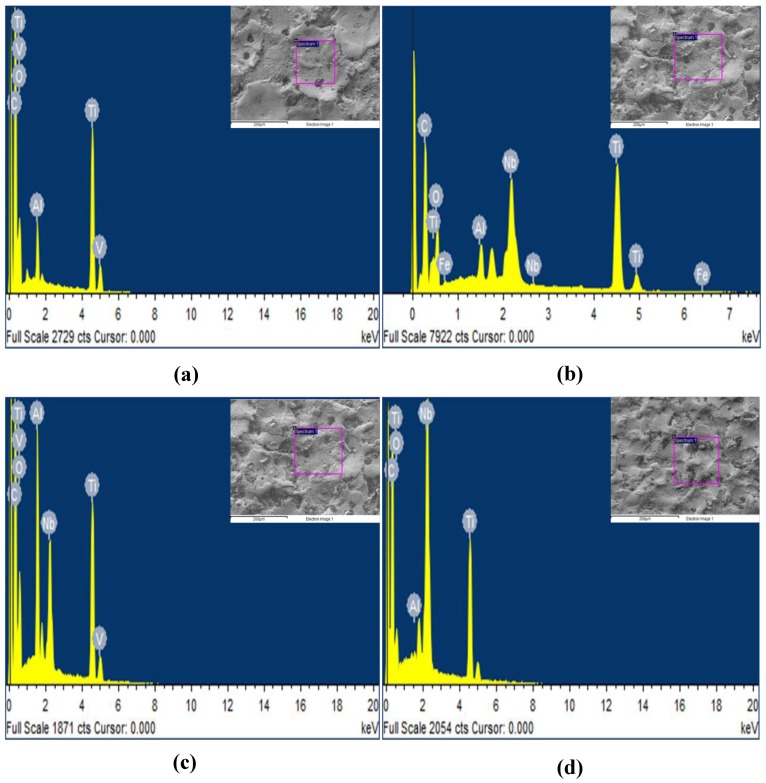
Energy dispersive X-ray spectra of EDM-treated (**a**), EDC-treated Nb coated surface at Nb concentration of (**b**) 10 g/L, (**c**) 15 g/L and (**d**) 20 g/L.

**Figure 9:**
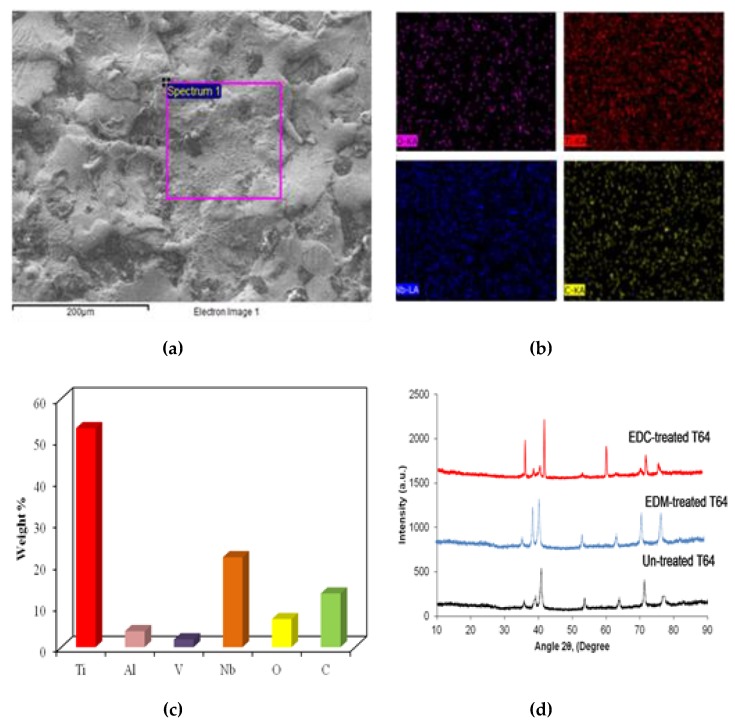
Mapping of EDC-treated Ti-6Al-4V alloy at peak current = 20 A, pulse duration = 250 µs, Duty cycle = 8%, and machining time = 60 min with Nb powder concentration of 20 g/L. (**a**) Spectrum area (**b**) Mapping of Ti, Nb, O and C (**c**) Weight Percentage of elements present in coated layer (**d**) XRD pattern of un-treated, EDM-teraed, and EDC-treated Ti-64.

Figure 8 shows the EDS spectrum of EDM-treated and EDC-treated of Ti-6Al-4V alloy setting the working condition of peak current = 20 A, pulse duration = 250 µs, duty cycle = 8%, and machining time = 15 min with Nb powder concentration of 0, 10, 15 and 20 g/L. The EDS results show that the EDM-treated surface has no significant presence of Nb powder particles (Figure 8a). On the other hand, the EDC-treated surface has found a significant amount of Nb powder with the weight percentages of 5.98%, 14.87%, and 21.47% as shown in Figure 8b–d. Furthermore, in addition to the presence of Nb, other elements like Ti, Al and, V along with O and C were also present on the EDC-treated surface. The micro-level mapping of the EDC-treated surface at the working condition of peak current = 20 A, pulse duration = 250 µs, duty cycle = 8%, and machining time = 60 min with Nb powder concentration of 20 g/L at a magnification of 250× is shown in Figure 9. The area selected for mapping is shown in Figure 9a. Figure 9b shows the maps of element Nb, Ti, O, and C, changes the surface composition and formed various oxides and carbides, which further increased the surface hardness of the coated layer. Figure 9c shows the composition of the coated surface with carbon of 12.97%, oxygen of 6.97%, niobium 21.67%, and titanium of 52.84%, respectively. The presence of elements O and C indicates the possible formation of oxides and carbides (Ti-C, Ti-O, Nb-C, and Nb-O). The peaks of the coated surface were allocated JCPDS reference nos: 03-065-5714 (TiO2), 00-002-0943 (TiC), JCPDS: 00-002-1031 (NbC), 00-017-0127 (NbC). Figure 9d shows the XRD pattern of un-treated, EDM-treated, and EDC-treated Ti-64 alloy.

**Figure 10:**
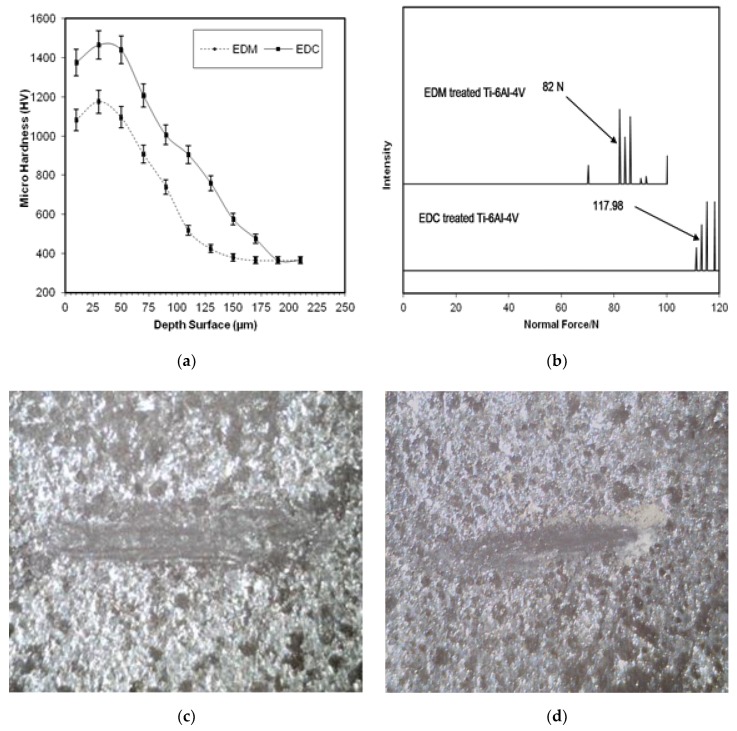
(**a**) Distribution of micro-hardness along the cross section, (**b**) adhesion strength of electric. discharge (ED)-coated and EDM-treated surface, and (**c**,**d**) scratch images on EDM-treated and EDC-treated surfaces.

Figure 10 shows the distribution of micro-hardness along the cross section of ED-coated and EDM-treated surface. The micro-hardness decreases gradually from the top surface to the base surface. The highest micro-hardness of 1465 HV appears at few microns away from the top surface in the case of EDC. The improvement in the microhardness is mainly attributed to the reinforcement effect of Nb/Ti-based oxides and carbides during the manufacturing process, which is transferred to the electrode and dielectric fluid. In the EDC process the resolidification of molten pool is very rapid and changes in the microstructure are evident. The EDC-modified layer exhibited various types of oxides and carbides (Ti-O, TiC, Nb-C, and Nb-O) with excellent metallurgical bond, which enhances the microhardness of the surface. Whereas, the highest microhardness of 1175 HV appears at few microns away from the top surface in the case of EDM. The microhardness of the EDM-treated surface is low as compared to the EDC-treated surface. This is because, during the EDM process at positive polarity, the high temperature leads to the phase transformation being impeded, producing a structure with micro-cracks with the low metallurgical bond. As a result of this, the mechanical properties weaken and the hardness value drops. The metallurgical bond establishes the adhesion strength. A stronger metallurgical bond promote a higher adhesion strength. Figure 10b shows the adhesion strength of the coating. It is clear that, the coated layer has excellent metallurgical bonding to the substrate surface. Therefore, no delamination of the coated layer was observed in both cases, but the EDM-treated surface is slightly affected in respect to the EDC-treated surface that perform well even at higher load. Figure 10c,d shows the EDM-treated and EDC-treated surfaces, respectively. The Nb-deposited surface possessed high surface hardness and offered excellent mechanical interlocking with the substrate. Benefitting from this mechanical interlocking, the critical adhesion failure of EDM-treated surface and Nb-deposited surface was detected at 82 N and 118 N respectively, as can be seen in Figure 10. This observation permits us to indicate the EDC-treated surface as suitable candidate counterpart for tribological properties.

## 4. Conclusions

This study focused on the application of spark plasma sintered Ti-Nb alloy under electric discharge coating in order to improve the surface charctristics of Ti-6Al-4V alloy using the Nb powder mixed dielectric and permits us to drawn the following conclusions:

1. A partially sintered Ti-Nb alloy has been successfully fabricated by mechanical alloying of Ti and Nb from powders, alloy modified later by spark plasma sintering technique, which was further used as tool electrode for the EDC process.
2. The surface of Ti-6Al-4V alloy has been modified by EDM (positive Polarity) and EDC (negative Polarity). The EDC-treated surface contains only few cracks and smooth geometry as compared to the EDM-treated surface.
3. A coating layer of Ti-O, TiC, Nb-C, and Nb-O have been successfully prepared by using partially sintered Ti-Nb alloy and Nb powder mixed in the dielectric fluid of electric discharge machine. The mass deposition of coating layer have almost linear relation and is significantly affected by the concentration of peak current, pulse duration, and Nb powder concentration.
4. The thickness of coating layer was significantly affected by the concentration of peak current, pulse duration, and Nb powder concentration. Using the 0 g/L Nb concentration (EDM), the coating layer thickness ~ 149 µm was obtained for a longer pulse duration (400 µs). On the other hand, when the concentration of Nb was increased to 10 g/L and 20 g/L, the thickness of the recast layer increased to 195 µm and 215 µm, respectively, in the same working conditions.
5. The EDS elemental map and XRD pattern analysis of the EDC-treated surface confirm the process in which the material migrate from the tool electrode to workpiece surface. Here, the suspended powder particles generated in the dielectric fluid which promote adhesion to the workpiece surface are playing the main role in the improvement of surface properties by generating favorable surface chemistry of oxides and carbides. When a higher concentration of Nb powder (20 g/L) is used, the EDC-treated surface is expected to form the Ti-O, Nb-O, Ti-C, and Nb-C like phases.
6. The surface coating layer permits an increase in the microhardness of the workpiece surface from 365 HV to 1465 HV and demonstrates an excellent metallurgical bonding with the base workpiece surface. The adhesion strength is as high as 118 N for the EDC-treated surface when compared to EDM treated surface at 82 N, respectively; thus indicating that the EDC-treated surface may have good tribological properties.
7. In summary, the EDC can be considered a great technique in order to improve surface characteristics and surface properties. The coating obtained by EDC process is more reliable and suitable for its purposes because the surfaces produced by this process demonstrated higher surface hardness that can be associated with better wear resistance of the implant. The Nb content in the coated layer provides superior corrosion resistance and allows improvements in the bioactivity of the implant substrate.

## Figures and Tables

**Table 1:** Experimental condition of electric discharge coating (EDC).

Name of Parameter	Range of Parameter
Workpiece	Ti-6Al-4V alloy
Tool electrode	CP-Ti alloy
Polarity	Reverse (−Ve) for EDC and Straight (+Ve) for electrical discharge machining (EDM)
Peak current	5, 10, 15, 20, 25 A
Pulse-on time	50, 100, 200, 400, 800 µs
Duty Cycle	8%
Dielectric medium	Hydrocarbon oil
Machining time	15 min
Powder Concentration	5, 10, 15, 20 g/L
